# Observational study on time on treatment with abiraterone and enzalutamide

**DOI:** 10.1371/journal.pone.0244462

**Published:** 2020-12-28

**Authors:** Giuseppe Fallara, Ingela Franck Lissbrant, Johan Styrke, Francesco Montorsi, Hans Garmo, Pär Stattin

**Affiliations:** 1 Division of Experimental Oncology/Unit of Urology URI, IRCCS Ospedale San Raffaele, Milan, Italy; 2 Vita‐Salute San Raffaele University, Milan, Italy; 3 Department of Surgical Sciences, Uppsala University, Uppsala, Sweden; 4 Department of Oncology Institute of Clinical Sciences, The Sahlgrenska Academy, University of Göteborg, Göteborg, Sweden; 5 Department of Surgical and Perioperative Sciences, Urology and Andrology, Umeå University, Umeå, Sweden; 6 Regional Cancer Centre, Uppsala/Örebro, Uppsala University Hospital, Uppsala, Sweden; Medizinische Universitat Innsbruck, AUSTRIA

## Abstract

**Introduction:**

The aim of this study was to assess time on treatment with abiraterone and enzalutamide, two androgen receptor targeted (ART) drugs, the impact on time on treatment of time interval without drug supply between prescription fillings, and adherence to treatment.

**Material and methods:**

By use of data from The National Prostate Cancer Register, The Prescribed Drug Registry and the Patient Registry, time on treatment with the abiraterone and enzalutamide was analyzed in all men with castration resistant prostate cancer (CRPC) in Sweden 2015–2019. Three time intervals between consecutive fillings, i.e. time without drug supply, were assessed. Adherence to the treatment was evaluated by use of the Medication Possession Ratio. Kaplan Meier analysis and multivariable Cox regression model were used to assess factors affecting time on treatment.

**Results:**

Between January 2015 and October 2019, 1803 men filled a prescription for abiraterone and 4 534 men filled a prescription for enzalutamide. With a time interval of 30 days or less between two fillings, median time on treatment was 4.9 months (IQR 2.6–11.7) for abiraterone and 8.0 months (IQR 3.6–16.4) for enzalutamide. In sensitivity analyses, allowing for no more than 14 days without drug supply between fillings, median time on treatment was 3.9 months (IQR 2.1–9.0) for abiraterone and 5.9 months (IQR 2.8–12.1) for enzalutamide. Allowing for any time period without drug between fillings, median time on treatment was 5.7 months (IQR 2.7–14.0) for abiraterone and 9.8 months (IQR 4.4–21.0) for enzalutamide. Adherence to treatment was above 90% for both drugs.

**Conclusion:**

Time on treatment with abiraterone and enzalutamide was shorter in clinical practice than in randomized controlled trials and varied almost two-fold with time interval without drug. Adherence to treatment was high. The main limitation of our study was the lack of data on use of chemotherapy.

## Introduction

Several randomized clinical trials (RCT) have shown that abiraterone and enzalutamide, two androgen receptor targeted (ART) drugs, increase survival and improve quality of life in men with metastatic castration resistant prostate cancer (mCRPC) [1–5]. Therefore, several national guidelines including those in Sweden, as well as guidelines from the European Association of Urology (EAU) and American Urological Association (AUA) recommend their use, either before or after chemotherapy [6–8]. In these RCTs, the median time on treatment was around 14 months for abiraterone and 17 months for enzalutamide in chemo-naïve men and around 8 months in post-docetaxel setting for both drugs. There has been concern that use of ART drugs in clinical practice is continued too long and given that these drugs are expensive and are used by a large number of men with mCRPC, this could have large financial implications. A wide range in time on treatment for these drugs has been reported in previous observational studies, ranging from 3 to 20 months (**S1 Table**) [9–25].

The aim of this study was to assess time on treatment for abiraterone and enzalutamide, the effect on time on treatment by the allowed time interval without drug between fillings and adherence to the treatment in a nationwide population-based study of men with mCRPC in Sweden.

## Materials and methods

### The National Prostate Cancer Register (NPCR) of Sweden

Since 1998, the NPCR includes data on 98% of all newly diagnosed cases of prostate cancer (PCa) registered in the Swedish Cancer Registry, to which reporting is mandatory by law [26,27]. Comprehensive data on cancer characteristics, work-up, and primary treatment are registered but no data on the subsequent disease trajectory are registered.

### Prostate Cancer data Base Sweden (PCBaSe)

In PCBaSe, NPCR has been linked to other national health care registers and demographic databases by use of the Swedish personal identification number. In this study we used data from PCBaSe RAPID 2018 that includes all men diagnosed with PCa until December 31, 2018 and with follow-up until October 31, 2019 [28].

### The Prescribed Drug Registry

The Prescribed Drug Registry comprises all filled prescribed drugs to the entire Swedish population since July 2005 [29]. It contains the following information: the Swedish person identity number, the dispensed drug name and ATC-code (Anatomical Therapeutic Chemical classification), the defined daily dose (DDD), the amount of drug dispensed and the date of dispensing. In this registry there are no data on drugs delivered in hospital or delivered over the counter without a prescription. We used The Prescribed Drug Registry to search for filled prescriptions for abiraterone and enzalutamide (ATC codes L02BX03 and L02BB04, respectively).

### The National Patient Registry

The National Patient Registry includes information regarding in-patient care in Sweden from 1987 and an increasing capture of out-patient care since 2000. This registry was used to compute the Charlson comorbidity index (CCI) based on discharge diagnoses up to 10 years prior to ART initiation, and to retrieve data for chemotherapy before ART initiation [30].

### The Cause of Death Registry

Since 1991, the Cause of Death Registry collects information on the date and cause of death for all persons registered in Sweden. The validity of data in this registry has been found to be high for PCa death [31].

### Subsidized use of abiraterone and enzalutamide

The Dental and Pharmaceutical Benefits Agency is a Swedish government agency whose remit is to decide which pharmaceutical products should be subsidized. Both abiraterone and enzalutamide are subsidized. The New Therapies council together with national guideline groups make recommendations and formulates follow-up protocols for new drug therapies after market authorization.

#### Study design and patient selection

This study included men in NPCR who were diagnosed with PCa until December 31, 2018 and who had filled a first prescription for abiraterone or enzalutamide in the Prescribed Drug Registry between July 1, 2015 and October 31, 2019. Data were accessed in May 2020 for analysis.

The only indication for abiraterone and enzalutamide was mCPRC until June 15, 2018 when abiraterone was licensed for men with metastatic hormone sensitive PCa [32]. Thus, we assumed that men who had filled a prescription for abiraterone or enzalutamide were in mCRPC state, with the exception of men who had filled a prescription for abiraterone less than six months after PCa diagnosis, who were excluded. The use of the first prescribed ART was assessed, i.e. time on treatment for abiraterone after initial use of enzalutamide and vice versa was not analyzed (**S1 Fig**).

We used data from PCBaSe on PCa risk category [26] and primary treatment, defined as curative (radical prostatectomy or radiotherapy) or non-curative (androgen deprivation therapy [ADT], active surveillance or deferred treatment), age at ART initiation, duration of ADT (defined as the time from the date of initiation of GnRH agonist/antagonist, bicalutamide or orchiectomy whichever came first, to the start date of ART), time from diagnosis to ART start, CCI, and previous chemotherapy.

#### Study outcomes

**Fig 1:**
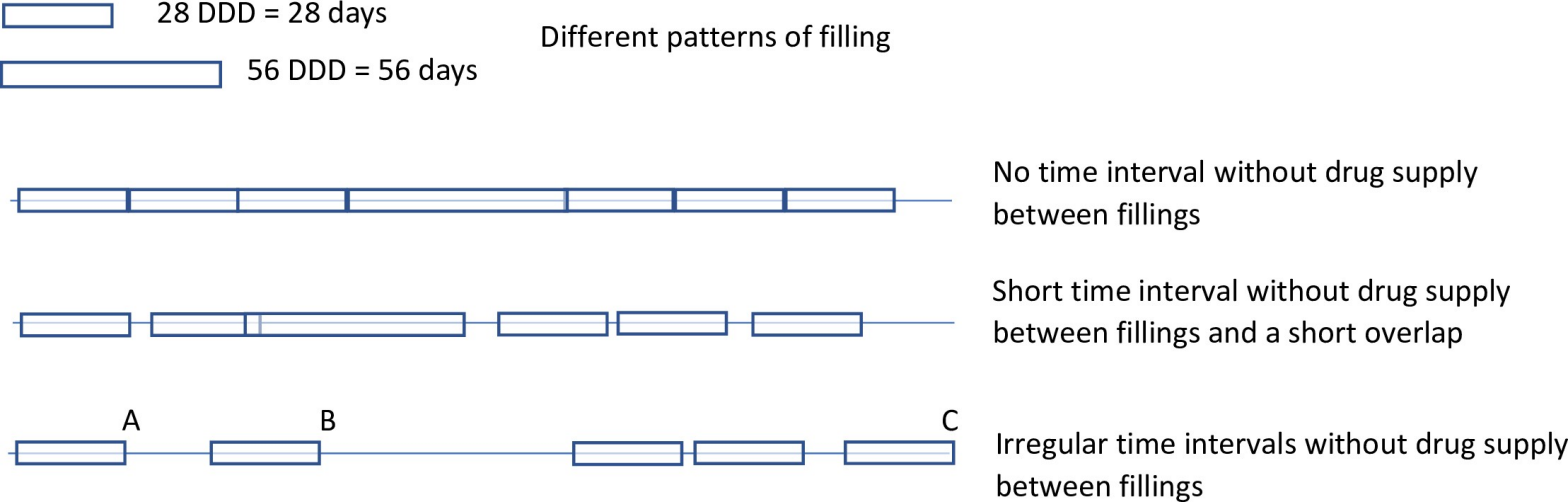
Possible scenarios for filled prescriptions in the Prescribed Drug Registry. Legend: A: Stop date if 14 days are allowed between period covered by drug supply. B: Stop date if 30 days are allowed between period covered by drug supply. C: Stop date if no restriction on time interval between fillings is applied. The Defined daily dose (DDD) corresponds to two pills per day for abiraterone and to one per day for enzalutamide, and a package usually supplies drugs for 28 days. There is sometimes a time period without drug supply. This time period allows for a putative immortal time bias. To limit this bias we used 30 days as the longest time period without drug supply (using the 14 days and no restriction as sensitivity analysis). DDD = Defined Daily Dose.

The primary outcome of the study was time on treatment for abiraterone and enzalutamide. Time on treatment started at the date of the first filling of the drug. To define end of time on treatment we used the defined daily dose (DDD) registered in the Prescribed Drug Registry. For both abiraterone and enzalutamide the registered DDD is the number of days for which the drug is supplied in the filling, which is usually 28 days. After DDD days from the filling, there is sometimes a time period before the date of the next filling, i.e. the patient does not fill the next prescription exactly DDD days after the previous filling which means that the patient is without drug supply **(Fig 1**). We allowed this time period to be 30 days. If this period was longer than 30 days, then the date of the end of supply of the previous filling was considered as end of treatment. If the date of end of supply was at a later calendar date than date of death, then the latter date was used as end of treatment.

By allowing for a time period beyond the last drug supply immortal time bias is introduced [33]. As a sensitivity analysis we allowed 14 days or shorter and we also analysed time on treatment without restriction on the time without supply. We also assessed adherence to treatment and predictors of treatment stop.

#### Statistical analysis

Cumulative incidence curves were created for time on treatment censoring at end of follow-up without an observed drug stop, considering death and other causes for treatment stop as competing risks. Median time on treatment was estimated by use of Kaplan Meier method, i.e. sum of cumulative incidence of death and drug stop.

Adherence was assessed by use of the Medical Possession Ratio (MPR), defined as the ratio between the sum of the total days of supply and the total number of days in the observation period [34]. Mean time on treatment was calculated as the area under the survival curve fitted by a Weibull distribution [35–37]. The product of adherence and mean time on treatment was used to estimate the duration of time on treatment covered by drug supply.

We combined the use of abiraterone and enzalutamide in order to assess factors that were associated with drug stop by use of Kaplan Meier plots and in univariable and multivariable Cox regression models.

The study was approved by the Research Ethics Board in Uppsala (PCBaSe RAPID 2018 Dnr 2017–263) that waived the informed consent requirement.

## Results

6 337 men had filled a prescription of ART, of whom 1 803 had filled a prescription for abiraterone and 4 534 for enzalutamide (**S1 Fig**).

At date of diagnosis, 751 men (42%) on abiraterone and 1 877 men (42%) on enzalutamide had metastatic disease. Accordingly, the majority of men had undergone non-curative primary treatment (**Table 1**).

**Table 1:** Baseline characteristic of 6 337 men in prostate cancer data base Sweden (PCBaSe) RAPID 2018 who had filled a prescription for abiraterone or enzalutamide in addition to GnRH.

	Abiraterone (n = 1 803)	Enzalutamide (n = 4 534)
**At date of diagnosis**		
**PCa risk category, n (%)**^**#**^		
Low/Intermediate-risk	373 (21)	834 (18)
High-risk/Locally advanced	669 (37)	1805 (40)
Metastatic	751 (42)	1877 (42)
Missing	10(0)	18 (0)
**Primary treatment, n (%)**^**$**^		
Non-curative	1260 (70)	3076 (68)
Curative treatment	482 (27)	1307 (29)
Missing	61 (3)	151 (3)
**PSA at diagnosis, ng/mL**		
Median (IQR)	31.4 (12–124)	33.0 (12–127)
Missing	12 (0.7)	36 (0.8)
**At date of initiation of ART**		
**Age, Years**		
Median (IQR)	76.7 (71.1–82.1)	76.1 (71–81.4)
**Calendar year of prescription, n (%)**		
2015	235 (13)	725 (16)
2016	245 (14)	1053 (23)
2017	390 (22)	930 (21)
2018	437 (24)	1049 (23)
2019*	496 (27)	777 (17)
**Time from diagnosis to ART start, n (%)**		
Median (IQR), years	5.6 (2.6–10.3)	5.5 (2.6–9.7)
0.5–1 year	84 (5)	212 (5)
1–3 years	446 (25)	1126 (25)
3–5 years	296 (16)	750 (16)
5–10 years	493 (27)	1379 (30)
>10 years	484 (27)	1067 (24)
**Time from start of ADT to start of ART, n (%)**		
≤ 0.5 year	34 (2)	77 (2)
0.5–1 year	89 (5)	253 (6)
1–3 years	535 (30)	1345 (29)
3–5 years	354 (20)	906 (20)
5–10 years	550 (30)	1114 (24)
>10 years	241 (13)	839 (18)
**Charlson comorbidity index, n (%)**		
0	644 (36)	1622 (36)
1–2	614 (34)	1488 (33)
3–4	366 (20)	907 (20)
≥ 5	179 (10)	517 (11)
**Previous chemotherapy, n (%)**		
Not recorded in PAR	1601 (89)	3999 (88)
Yes	202 (11)	535 (12)

^#^ PCa risk category definitions: Low-risk: T1-2, Gleason score 2–6 and PSA <10 ng/ml; Intermediate-risk: T1-2, Gleason score 7 and/or PSA 10 to <20 ng/ml; High-risk: T3 and/or Gleason score 8–10 and/or PSA 20 to <50 ng/ml; Locally advanced: T4 and/or N1 and/or PSA 50 to <100 ng/ml in the absence of distant metastases (M0 or Mx); Metastatic: M1 and/or PSA ≥100 ng/ml.

^$^ Primary treatment was defined as: curative: radical prostatectomy or prostate radiotherapy; non-curative: active surveillance, watchful waiting or primary androgen deprivation therapy.

* Data available until 2019-10-31.

Legend: IQR: interquartile range, PCa: prostate cancer; ART: androgen receptor targeted therapy; ADT: androgen deprivation therapy; PAR: the In-Patient Registry.

Median age at drug initiation was 77 years (interquartile range [IQR] 71–82) for abiraterone and 76 years (IQR 71–81) for enzalutamide. Median time from diagnosis to start of treatment was 5.5 years for both abiraterone and enzalutamide. Time from ADT start to abiraterone or enzalutamide start was more than 5 years in almost 40% of men and less than 1 year in less than 10% of men. The majority of men, almost 70%, had CCI ≤ 2.

### Median time on drug according to different time intervals without drug supply

**Fig 2:**
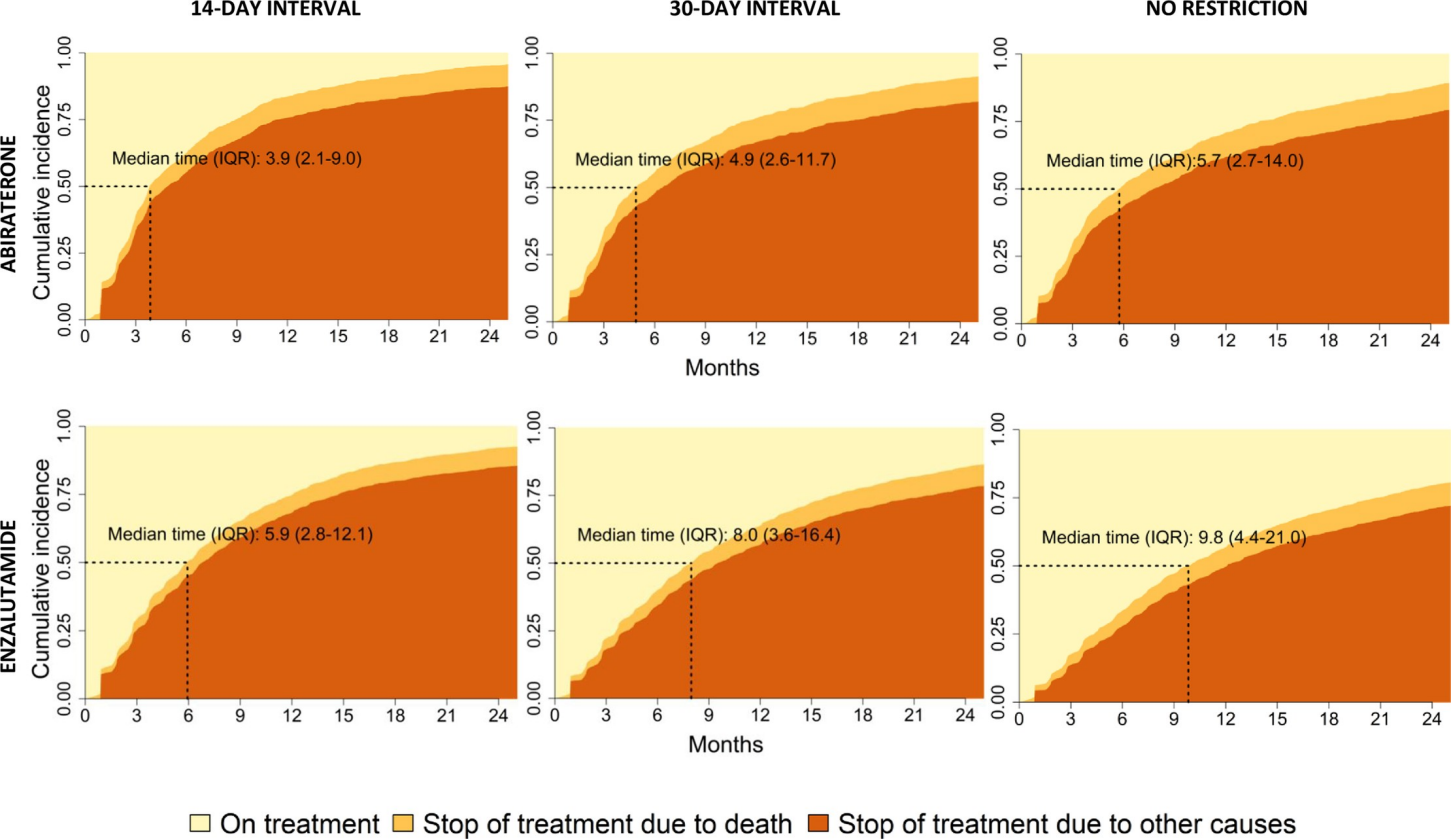
Time on treatment with abiraterone and enzalutamide. Legend: Cumulative incidence of drug stop using time interval between fillings of 14-day, 30-day or no restriction. For definition of drug stop see Fig 1.

Median time on treatment was 4.9 months (IQR 2.6–11.7) for abiraterone and 8.0 months (IQR 3.6–16.4) for enzalutamide, using 30 days or less as time interval between two consecutive fillings without drug supply (**Fig 2**). We also assessed shorter and longer time intervals between two fillings. For a shorter time interval, up 14 days without drug supply, median time on treatment was shorter, 3.9 months (IQR 2.1–9.0) for abiraterone and 5.9 months (IQR 2.8–12.1) for enzalutamide. For a longer time interval without drug supply, allowing for any time period without drug before the next filling, median time on treatment became longer, 5.7 months (IQR 2.7–14.0) for abiraterone and 9.8 months (IQR 4.4–21.0) for enzalutamide.

### Adherence

Adherence assessed as Medication Possession Ratio was 97% for abiraterone and 95% for enzalutamide. Less than 5% of men on abiraterone and 8% on enzalutamide had low adherence (< 80%). By use of a time interval of 30 days, the duration of mean time on treatment covered by drug was 8.5 months for abiraterone and 11.6 months for enzalutamide **(S2 Fig)**.

### Factors affecting time on treatment

**Fig 3:**
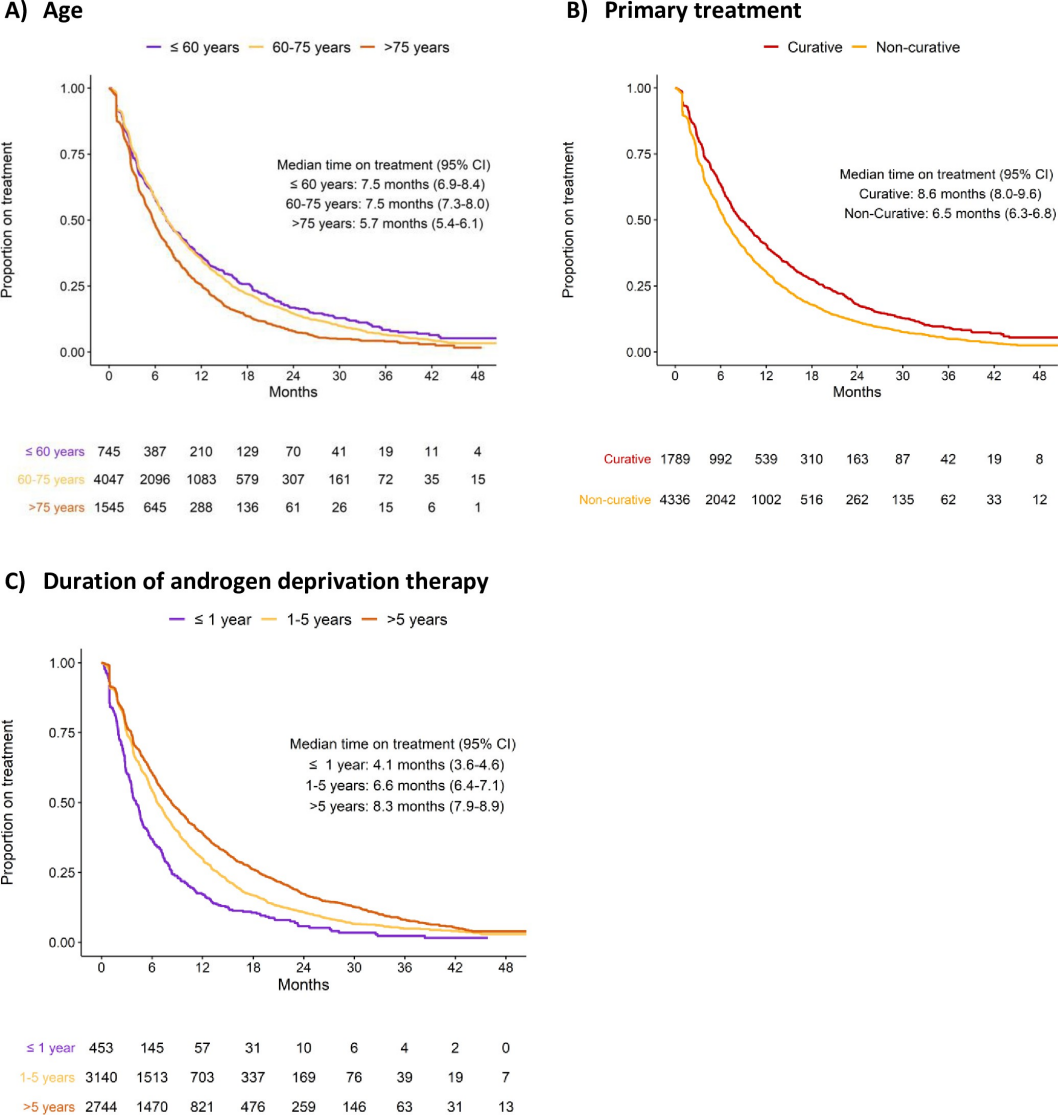
Time on treatment stratified by age at drug initiation (A), primary treatment (B), and androgen deprivation therapy duration before ART (C).

A shorter time on treatment was found in old men, in men who had receive non-curative primary treatment, in men with high comorbidity, and in men with short time on ADT prior to ART **(Fig 3)**. These findings remained after adjustments in the multivariable Cox regression analysis **(Table 2)**.

**Table 2:** Risk of treatment stop according to age, comorbidity, cancer characteristics at diagnosis and cancer treatments.

		Univariable HR (95% CI)	Multivariable HR (95% CI)
**Age at diagnosis**	≤ 60 years	1.00 (ref)	1.00 (ref)
	60–75 years	1.07 (0.97, 1.17)	0.98 (0.89, 1.08)
	>75 years	1.41 (1.27, 1.56)	1.15 (1.03, 1.28)
**Charlson comorbidity index**	0	1.00 (ref)	1.00 (ref)
	1–2	1.06 (0.99, 1.13)	1.06 (0.98, 1.13)
	≥ 3	1.30 (1.21, 1.39)	1.29 (1.20, 1.38)
**PCa risk category at diagnosis**^**#**^	Low/Intermediate-risk	1.00 (ref)	1.00 (ref)
	High-risk/Locally advanced	1.02 (0.94, 1.10)	0.99 (0.91, 1.07)
	Distant metastasis	1.17 (1.08, 1.27)	0.95 (0.87, 1.04)
**Primary treatment**^**$**^	Curative	1.00 (ref)	1.00 (ref)
	Non-curative	1.32 (1.24, 1.41)	1.22 (1.13, 1.32)
Duration of androgen deprivation therapy*	≤1 year	1.00 (ref)	1.00 (ref)
	1–5 years	0.69 (0.62, 0.77)	0.71 (0.63, 0.79)
	>5 years	0.55 (0.49, 0.61)	0.58 (0.52, 0.66)

Univariable and multivariable Cox regression model assessing the risk of drug stop including as covariates age at diagnosis, Charlson comorbidity index, PCa risk category, primary treatment at diagnosis and duration of androgen deprivation therapy.

^#^ PCa risk category definitions: Low-risk: T1-2, Gleason score 2–6 and PSA <10 ng/ml; Intermediate -risk: T1-2, Gleason score 7 and/or PSA 10 to <20 ng/ml; High-risk: T3 and/or Gleason score 8–10 and/or PSA 20 to <50 ng/ml; Locally advanced:T4 and/or N1 and/or PSA 50 to <100 ng/ml in the absence of distant metastases (M0 or Mx); Metastatic: M1 and/or PSA ≥100 ng/ml.

^$^ Primary treatment was defined as: curative: Radical prostatectomy or prostate radiotherapy; non-curative: Active surveillance, watchful waiting or primary androgen deprivation therapy.

* Duration of androgen deprivation therapy was defined as the time from the date of initiation of GnRH agonist/antagonist, bicalutamide or orchiectomy whichever came first, to the start date of ART.

HR: Hazard ratio; CI: Confidence interval; PCa: Prostate cancer.

Men who received ART after chemotherapy had a shorter time on treatment than men who received ART before chemotherapy, however only 12% of men were reported to have received chemotherapy (**S3 Fig**).

## Discussion

In this population-based, nationwide registry study, time on treatment in clinical practice was 5 months for abiraterone and 8 months for enzalutamide for 30 days or less without supply between two fillings. Adherence to treatment was above 90% for both abiraterone and enzalutamide.

### Strengths and limitations

The main strength of this study is the virtually complete capture of all men in a country on abiraterone or enzalutamide in 2015–2019. In addition, we had access to data that allowed us to assess time on treatment according to different time intervals, i.e. time without drug supply between fillings.

However, this study is not devoid of limitations. We did not have data on PSA levels that confirmed the diagnosis of CRPC. However, since the only indication for abiraterone and enzalutamide during the study period was mCRPC we argue that men who filled prescriptions for these drugs had reached the mCRPC state.

In absence of ECOG score, we used CCI to assess general health. CCI has previously been demonstrated to predict overall survival in PCa men quite well in NPCR [38]. A main limitation of our study is also the poor capture of chemotherapy in The In-Patient Registry [39]; only 12% of the men were recorded to have received chemotherapy. In contrast, in a previous report with data from pharmacies on in-patient treatment with chemotherapy between 2008 and 2010 21% of men who died for PCa had received chemotherapy before date of death, and we assume that a substantially higher proportion would have received chemotherapy in this more recent calendar period [40]. Finally, a limitation is the lack of information on the reason for drug stop.

### Time on treatment in previous published studies

Time on treatment in our study was considerably shorter than those in RCTs, which was around 14 months for abiraterone and 17 months for enzalutamide in chemo-naïve men and around 8 months post-docetaxel for both the drugs [1–4]. Of note, men in our study were considerably older at ART start and had more comorbidities than men in these RCTs. Our findings are in line with a majority of previous registry studies, where time on treatment ranged from 4 to 9 months for abiraterone and from 3 to 11 months for enzalutamide [9–12,16]. Finally, several single and multi-institutional observational studies have reported time on treatment for ART with a range from 3 up to more than 20 months, likely due to differences in study design, study population, and definition of drug stop [13–15,17–25].

### Implications of time interval between fillings on time on treatment

No consensus exists on what time interval between fillings should be used when assessing time on treatment for ART drugs from drug registries. In previous registry studies there has been a large range in the allowed time interval without drug supply from 45 to 90 days [9–12,16]. However, as the time interval between two fillings increases, the risk of immortal time bias increases, whereas a short time interval will underestimate time on treatment [41]. Immortal time bias in pharmaco-epidemiological studies refers to a period when follow-up cannot occur [33]. This is the case for a time interval without drug supply between two consecutive fillings: treatment stop cannot occur, but that time is included in time on treatment. In addition, immortal time bias is introduced because only men surviving long enough could have a drug stop date beyond a long interval in drug supply. We assessed time on treatment by use of three different time intervals between two consecutive fillings. Time on treatment increased almost 50% when this interval was allowed to increase from 14 days to no limit. These results underline the importance of reporting the time interval without drug supply in observational studies. However, there was no information on this time interval in 13/17 observational studies that were reviewed (**S1 Table**). We argue for the use of a 30-day limit for the time interval without drug supply for ART drugs as a reasonable compromise to be applied in observational studies based on data in Prescribed Drug Registries.

### Median and mean time on treatment and adherence

Mean time on treatment was longer than median time on treatment for both abiraterone and enzalutamide, which shows that some men use ART drugs for an extended time, e.g. 10% of men had a time on treatment longer than 23 months for abiraterone and 30 months for enzalutamide. Mean time on treatment is a reliable measure of the average amount of drug used, since it accounts for men with very long or short time on treatment better then median treatment duration [35–37].

In line with previous reports, adherence to the treatment with abiraterone and enzalutamide was above 90% for both the drugs [42–44], likely due to that there is a low risk of short term serious adverse events for men on ART drugs [45].

### Predictors of drug stop

In line with previous findings, time on treatment was shorter in old men, in men who had received primary non-curative treatment, in men with a short time on ADT before start of ART, and in men with high comorbidity [46].

## Conclusion

In this register-based study of all Swedish men with mCRPC in 2015–2019, time on treatment was 5 months for abiraterone and 8 months for enzalutamide. The concern that men with mCRPC continue to use ART drugs for too long in clinical practice was not substantiated in our study. Adherence to treatment was high. Men with aggressive prostate cancer had shorter time on treatment.

## Supporting information

S1 Fig Flow diagram with patient selection for abiraterone and enzalutamide groups. (DOCX)

S2 Fig Adherence to abiraterone and enzalutamide by use of Kaplan-Meier and Weibull curve according to 30-day time interval. (DOCX)

S3 Fig Time on treatment with abiraterone and enzalutamide in men with or without prior chemotherapy. (DOCX)

S1 Table Studies reporting time on treatment with abiraterone acetate and enzalutamide. (DOCX)

S1 File Literature search. (DOCX)
