# Supplementary material for: Observational study on time on treatment with abiraterone and enzalutamide
Source: PLoS One. 2020 Dec 28;15(12):e0244462. doi: 10.1371/journal.pone.0244462 (PMC7769419; doi:10.1371/journal.pone.0244462)
Supplement: S1 Fig — (DOCX) [file pone.0244462.s001.docx]

**7489** men in NPCR filled a prescription for abiraterone and/or enzalutamide in the Prescribed Drug Registry

**4534** men filled one or more prescriptions for enzalutamide

Men excluded:

- **280** = first prescription before 2015-07-01
- **781** = enzalutamide before abiraterone and vice versa
- **91** = first prescription within 6 months from diagnosis

**1803** men filled one or more prescriptions for abiraterone

**Supplementary Figure 1 - Flow diagram of selection of study men on abiraterone or enzalutamide in PCBaSe**

*NPCR = National Prostate Cancer Register of Sweden*
