# Supplementary material for: Observational study on time on treatment with abiraterone and enzalutamide
Source: PLoS One. 2020 Dec 28;15(12):e0244462. doi: 10.1371/journal.pone.0244462 (PMC7769419; doi:10.1371/journal.pone.0244462)
Supplement: S2 Fig — (DOCX) [file pone.0244462.s002.docx]

| **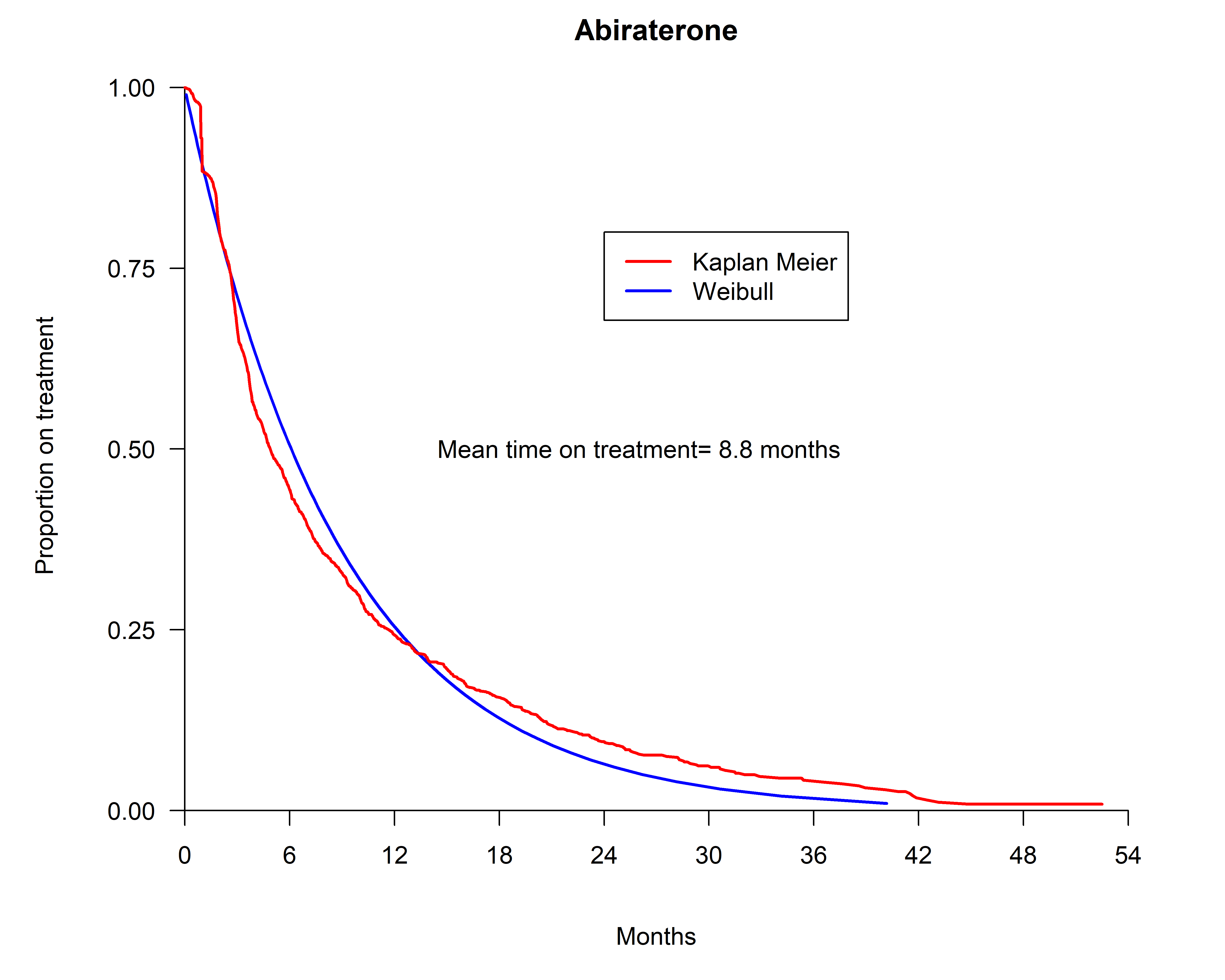** | **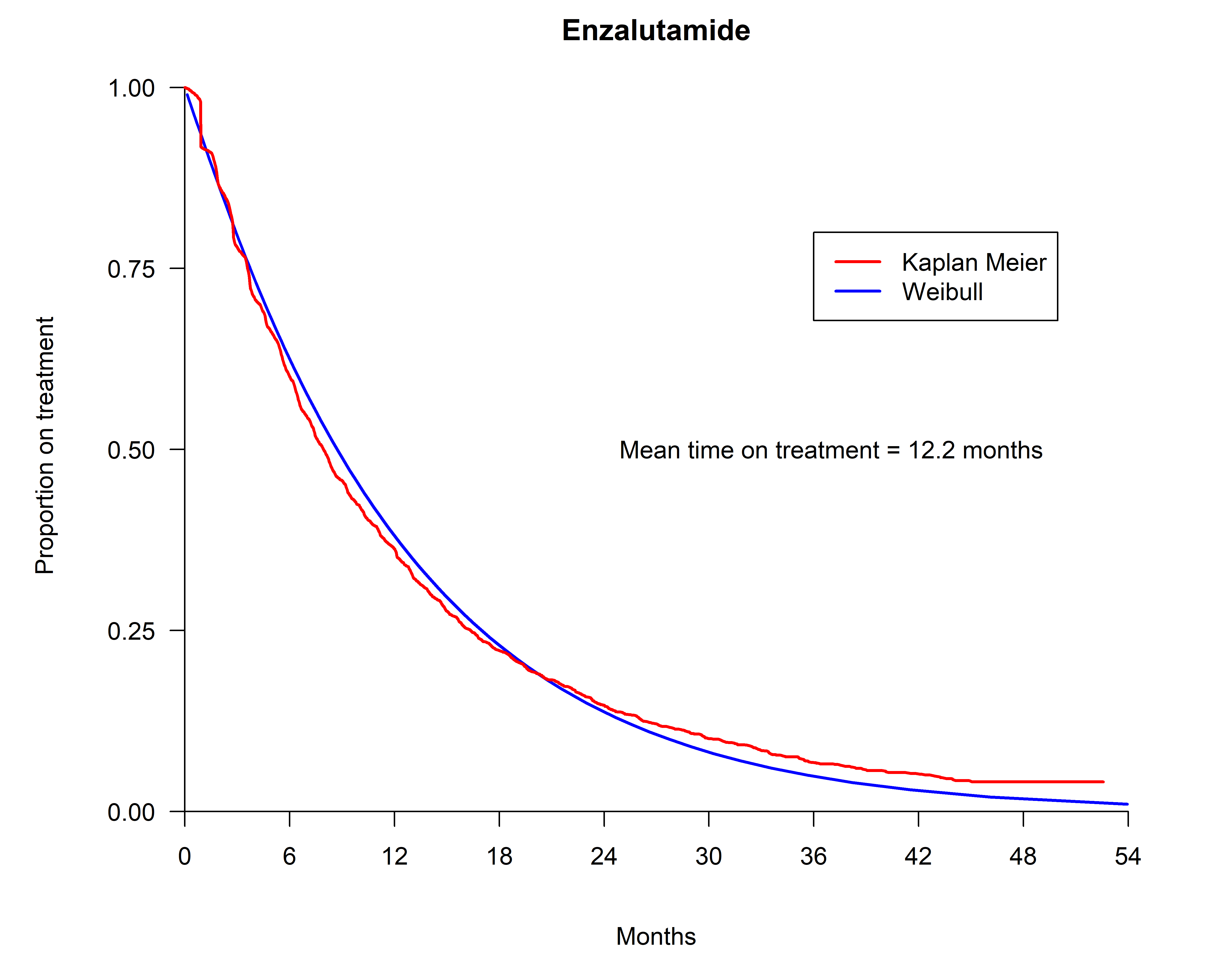** |
| --- | --- |

**Supplementary Figure 2 – Time on treatment with abiraterone and enzalutamide with fitted Weibull distribution using 30-day time interval between fillings**

*Mean time on treatment was calculated as the area under the fitted Weibull distribution survival curve.*
