# Supplementary material for: Observational study on time on treatment with abiraterone and enzalutamide
Source: PLoS One. 2020 Dec 28;15(12):e0244462. doi: 10.1371/journal.pone.0244462 (PMC7769419; doi:10.1371/journal.pone.0244462)
Supplement: S3 Fig — (DOCX) [file pone.0244462.s003.docx]

**Supplementary Figure 3-** Median time on treatment for abiraterone and enzalutamide in men with or without prior chemotherapy

*
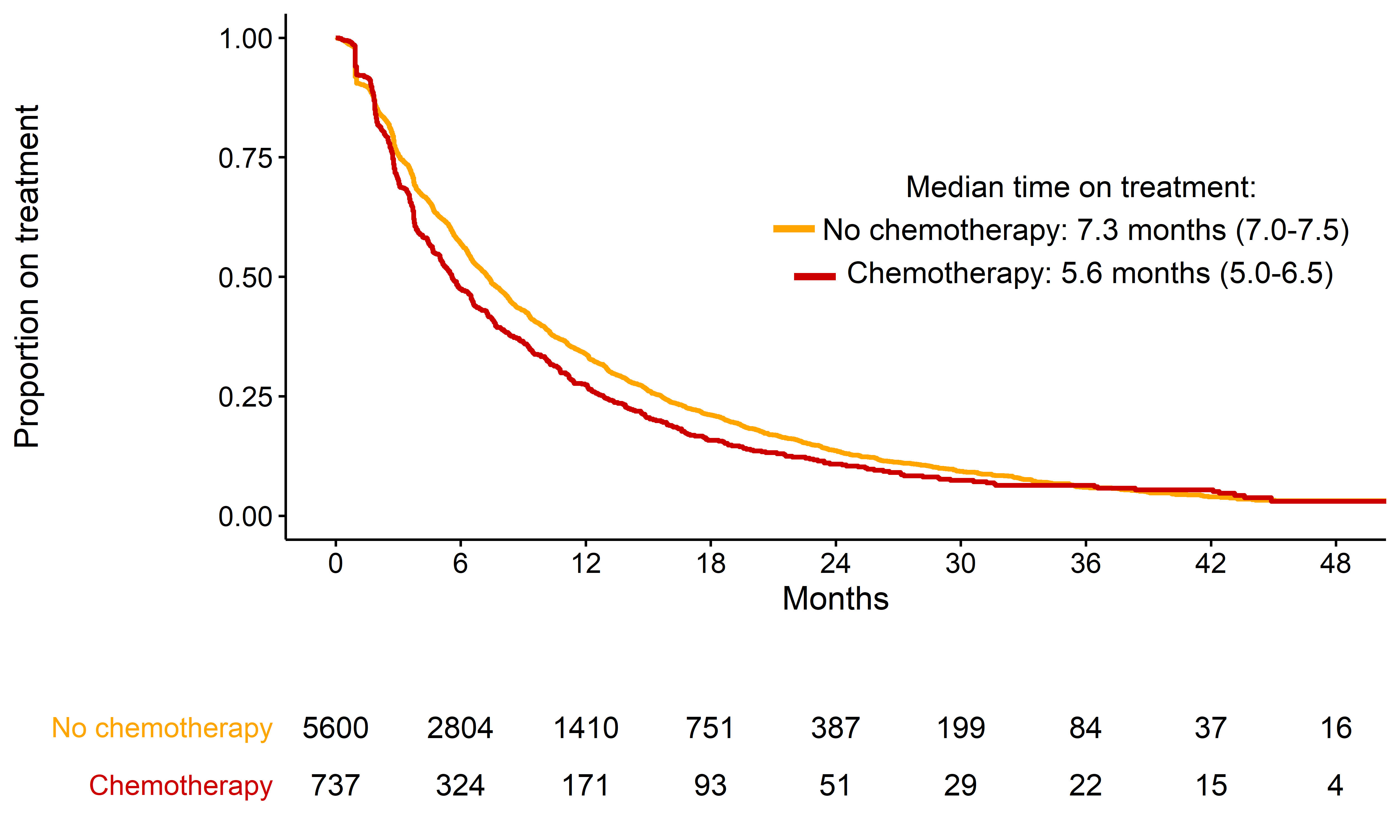
*

*Legend: chemotherapy according to data in The Patient Registry*
