# Supplementary material for: Observational study on time on treatment with abiraterone and enzalutamide
Source: PLoS One. 2020 Dec 28;15(12):e0244462. doi: 10.1371/journal.pone.0244462 (PMC7769419; doi:10.1371/journal.pone.0244462)
Supplement: S1 Table — (DOCX) [file pone.0244462.s004.docx]

**Supplementary Table 1 – Studies reporting time on treatment for abiraterone acetate and enzalutamide**

| **Year of publication** | **Author** | **Study design/ setting** | **Drug used** | **Setting (after /before chemo)** | **N of patient** | **Time interval between fillings** | **Median time on treatment** |
| --- | --- | --- | --- | --- | --- | --- | --- |
| **Randomized control trials** | | | | | | | |
| 2011 | De Bono et al. | RCT | AA | After chemo | 797 AA  398 placebo |  | **8** *months* |
| 2012 | Scher et al. | RCT | ENZ | After chemo | 800 ENZ  399 placebo |  | **8.3** *months* |
| 2015 | Rayan et al. | RCT | AA | Before chemo | 546 AA  542 placebo |  | **13.8** *months* |
| 2014 | Beer et al. | RCT | ENZ | Before chemo | 872 ENZ  845 placebo |  | **16.6** *months* |
| **Observational studies** | | | | | | | |
| 2014 | Azad et al. | Cancer registry | AA | Before chemo and after chemo | 519 | NA | **7.4** *months* ECOG 0-1  **4.5** *months* ECOG$\geq$2 |
| 2016 | Flaig et al. | Cancer registry | AA/ENZ | Before chemo | 3437 | 90 days | **4.25** *months* AA 1^st^ line  **4.25** *months* AA 2^nd^ line  **3** *months* ENZ 1^st^ line  **3.5** *months* ENZ 2^nd^ line |
| 2017 | Pilon et al. | Cancer registry | AA/ENZ | Before chemo and after chemo | 2,591 AA  807 ENZ | 60 days | **9.9** *months* ABI  **8.3** *months* ENZ |
| 2018 | Schultz et al. | Cancer registry | AA/ ENZ | Before chemo | 920 ENZ  2310 AA | 45 days | **8.8** *months* AA  **10.7** *months* ENZ  (sensitivity analysis with 30-day time interval: **8.0** vs. **8.3** and with 60-day time interval: **11.6** vs **10.2** months) |
| 2020 | George DJ et al. | Cancer registry | AA/ENZ | Before chemo and after chemo | 2559 | 90 days | **5.4** *months* AA 1^st^ line  **5.8** *months* ENZ 1^st^ line  **4.8** A *months* A 2^nd^ line  **5.4** *months* ENZ 2^nd^ line |
| 2015 | Houede et al. | Multi-center | AA | After chemo | 408 | NA | **5.2** *months* |
| 2016 | Svensson et al. | Multi-center | AA | After chemo | 119 | NA | **5.6** *months* |
| 2016 | Verzoni et al. | Multi-center | AA | After chemo | 148 | NA | **19.8** *months* |
| 2018 | Harshman et al. | Multi-center | AA | Before chemo and after chemo | 494 | NA | **14.2 -** **9.6** *months* I center (statin vs non statin)  **8.3 - 8** *months* II center (statin vs non statin) |
| 2019 | Boegemann M. et al. | Multi-center | AA | Before chemo | 480 | NA | **10.0** *months* |
| 2020 | Lu et al. | Multi-center | AA/ENZ | Before chemo and after chemo | 266 | NA | **7.1** or **10.3** *months* according to HSD3B1 mutations |
| 2014 | Leibowitz-Amid et al. | Single-center | AA | Before chemo and after chem | 111 | NA | **6.1** *months* overall  **7.4** *months* before chemo |
| 2017 | Ramalingam et al. | Single-center | AA | Before chemo | 135 | NA | **9.4** *months* White  **8.3** *months* Afro-American |
| 2017 | Salem et al. | Single-center | AA/ENZ | Before chemo | 76 AA  113 ENZ | NA | **10** *months* AA  **12** *months* ENZ |
| 2018 | Biró et al. | Single-center | AA | After chemo | 113 | NA | **6.4** *months* |
| 2019 | Beardo et al. | Single-center | AA/ENZ | Before chemo | 123 overall | NA | **16.6** *months* |
| 2019 | Alghazali et al. | Single-center | ENZ | Before chemo and after chemo | 102 before chemo  98 after chemo | NA | **13.8** *months* before chemo  **7.6** *months* after chemo |

*RCT: Randomized Control Trials; ECOG: Eastern Cooperative Oncology Group Performance status; chemo: chemotherapy; NA: not available; AA: abiraterone; ENZ: enzalutamide*

^#^ For search criteria and study selection see **Supplementary Material 1**
