# Supplementary material for: Observational study on time on treatment with abiraterone and enzalutamide
Source: PLoS One. 2020 Dec 28;15(12):e0244462. doi: 10.1371/journal.pone.0244462 (PMC7769419; doi:10.1371/journal.pone.0244462)
Supplement: S1 File — (DOCX) [file pone.0244462.s005.docx]

**Supplementary Material 1 - LITTERATURE SEARCH:**

 Inclusion criteria:

1. Subject: treatment duration of abiraterone and enzalutamide in CRPC patients (before or after chemotherapy)
2. Study design: randomized control trials, population database or cancer registries, single or multi-center studies.
3. Language: English
4. Period: form May 2011 – June 2020

Exclusion criteria:

1. Non-English articles
2. Before May 2011
3. Letter to the editor comment, editorial, abstracts, review and metanalysis
4. No full text availability
5. No reporting of treatment duration
6. Less than 100 patients

Search criteria in MEDLINE (through PubMed):

(“abiraterone” OR “enzalutamide”) AND ("prostate cancer” OR castra* OR CRPC) AND ("drug survival" OR "treatment duration") in the title + additional manual search

**21 reports identified**

**27 reports excluded after abstract or full text review**

**42 reports identified throughout the database searching**

**6 additional records through manual search**

**48 reports identified**

**Figure**: Flow diagram of included studies in literature review
